# Combination of CT and telomerase^+^ circulating tumor cells improves diagnosis of small pulmonary nodules

**DOI:** 10.1172/jci.insight.148182

**Published:** 2021-06-08

**Authors:** Wen Zhang, Xinchun Duan, Zhenrong Zhang, Zhenrong Yang, Changyun Zhao, Chunzi Liang, Zhidong Liu, Shujun Cheng, Kaitai Zhang

**Affiliations:** 1 Department of Immunology, National Cancer Center/National Clinical Research Center for Cancer/Cancer Hospital, Chinese Academy of Medical Sciences and Peking Union Medical College, Beijing, China.; 2 Department of Thoracic Surgery, Beijing Friendship Hospital, Capital Medical University, Beijing, China.; 3 Department of General Thoracic Surgery, China-Japan Friendship Hospital, Beijing, China.; 4 State Key Laboratory of Molecular Oncology, Department of Etiology and Carcinogenesis, National Cancer Center/National Clinical Research Center for Cancer/Cancer Hospital, Chinese Academy of Medical Sciences and Peking Union Medical College, Beijing, China.; 5 Chongqing Deepexam Biotechnology Co. Ltd., Chongqing, China.; 6 Wuhan Clinical Laboratory Center, Wuhan, China.; 7 Department of Thoracic Surgery, Beijing Chest Hospital, Capital Medical University, Beijing, China.

**Keywords:** Oncology, Pulmonology, Diagnostics, Lung cancer

## Abstract

**BACKGROUND:**

Early diagnosis and treatment are key to the long-term survival of lung cancer patients. Although CT has significantly contributed to the early diagnosis of lung cancer, there are still consequences of excessive or delayed treatment. By improving the sensitivity and specificity of circulating tumor cell (CTC) detection, a solution was proposed for differentiating benign from malignant pulmonary nodules.

**METHODS:**

In this study, we used telomerase reverse transcriptase–based (TERT-based) CTC detection (TBCD) to distinguish benign from malignant pulmonary nodules < 2 cm and compared this method with the pathological diagnosis as the gold standard. FlowSight and FISH were used to confirm the CTCs detected by TBCD.

**RESULTS:**

Our results suggest that CTCs based on TBCD can be used as independent biomarkers to distinguish benign from malignant nodules and are significantly superior to serum tumor markers. When the detection threshold was 1, the detection sensitivity and specificity of CTC diagnosis were 0.854 and 0.839, respectively. For pulmonary nodules ≤ 1 cm and 1–2 cm, the sensitivity and specificity of CTCs were both higher than 77%. Additionally, the diagnostic ability of CTC-assisted CT was compared by CT detection. The results show that CT combined with CTCs could significantly improve the differentiation ability of benign and malignant nodules in lung nodules < 2 cm and that the sensitivity and specificity could reach 0.899 and 0.839, respectively.

**CONCLUSION:**

TBCD can effectively diagnose pulmonary nodules and be used as an effective auxiliary diagnostic scheme for CT diagnosis.

**FUNDING:**

National Key Research and Development Project grant nos. 2019YFC1315700 and 2017YFC1308702, CAMS Initiative for Innovative Medicine grant no. 2017-I2M-1-005, and National Natural Science Foundation of China grant no. 81472013.

## Introduction

Lung cancer is the most common cancer worldwide in both incidence and mortality. It seriously affects public health and increases the socioeconomic burden. Early diagnosis and early treatment are key to the long-term survival of patients with lung cancer (1). The 5-year survival rate of patients with early lung cancer after surgical resection can reach 90% (2). However, early lung cancer mainly manifests as solitary pulmonary nodules (SPNs) in the lungs, which are mainly detected based on screening because they have no symptoms. The National Lung Screening Trial (NLST) shows that low-dose helical CT (LDCT) provides greater sensitivity for the early detection of lung cancer and reduces the risk of lung cancer mortality by 20% compared with chest radiography (3). Although 24% of participants were positive after LDCT screening, the benign proportion was as high as 96.4% after follow-up (3, 4).

At present, the clinical diagnosis of benign and malignant pulmonary nodules is mainly based on their size, density, morphology, composition ratio, and signs on CT. However, due to the diversity of pulmonary nodules, it is often difficult to distinguish benign and malignant diseases on CT. Positron emission tomography–CT (PET-CT) and CT-guided percutaneous biopsy are the methods used to further differentiate small lung nodules that are difficult to diagnose. Alternatively, the growth changes of these nodules can be followed up, and time is considered to be the best diagnostic approach. Although these treatment strategies follow guidelines (5–7), long waiting times cause anxiety in patients, with the attendant consequences of excessive or delayed treatment. Studies have shown that the number of benign pulmonary nodules after surgical treatment can be as high as 20% (8). Therefore, it is very important to accurately differentiate benign from malignant pulmonary nodules.

Liquid biopsy is a potentially novel and noninvasive method. Compared with traditional invasive biopsy, liquid biopsy has the advantages of better compliance, easy specimen acquisition, repeatability, and comprehensive tumor information. Therefore, it is expected to be an ideal technology for early auxiliary diagnosis, concomitant diagnosis, therapeutic monitoring, and prognostic assessment of cancer (9). The liquid part of liquid biopsy includes circulating tumor DNA (ctDNA), circulating tumor cells (CTCs), and exosomes. In the last 10 years, some studies have used CellSearch, folic acid receptor detection, and other detection methods based on CTCs to identify benign and malignant pulmonary nodules (10, 11), and some achievements have been made. However, there are still deficiencies in the sensitivity and specificity for the identification of smaller pulmonary nodules. Improving the sensitivity and specificity of CTC detection is also key to the identification of benign and malignant pulmonary nodules. Owing to CTC heterogeneity, the separation methods based on CTC surface biomarkers have some limitations (12). Therefore, other methods are needed to improve the detection ability of CTCs. In a previous study (13), we have reported a telomerase reverse transcriptase–based (TERT-based) CTC detection (TBCD) method. TERT is the basis for tumors to maintain their limitless replication potential, and upregulation of TERT activity has been detected in 80%–90% of malignancies compared with most normal cells, which lack telomerase activity (14, 15). TERT has also emerged as a potential diagnostic marker for tumors (16). The pathological type of most malignant lung nodules is adenocarcinoma, in which there is significant activity related to high TERT expression. Accordingly, there is intense interest and intuitive appeal in the discrimination of benign from malignant small pulmonary nodules with TBCD.

In this study, we used TBCD to detect CTCs in the peripheral blood (PB) of patients with pulmonary nodules within 2 cm in size, combined with tumor markers to assist comprehensive CT judgment, which improved the benign and malignant differential diagnosis of pulmonary nodules and helped patients benefit from diagnosis and treatment.

## Results

### Patient characteristics.

**Figure 1:**
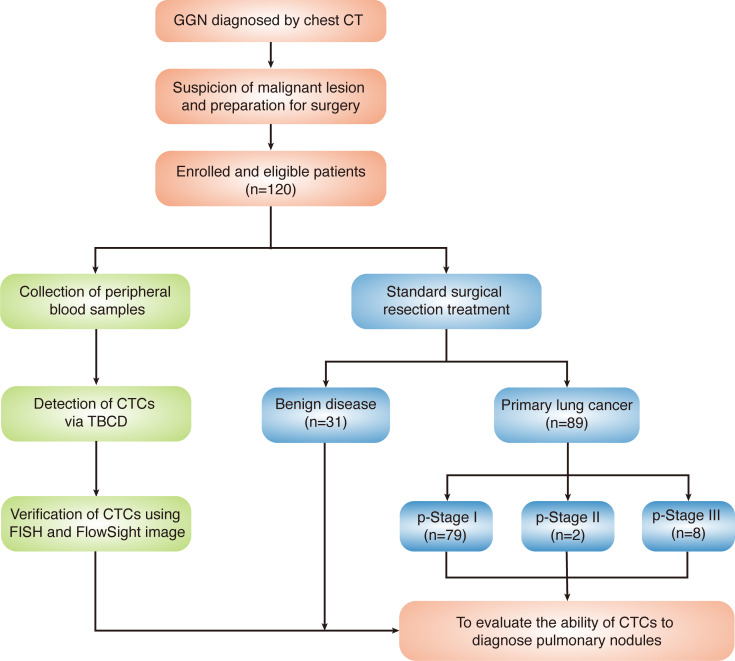
Flowchart of the diagnoses in the patients enrolled in this study. GGN, ground-glass nodules; p-stage, pathological stage.

From May 2017 to March 2019, consecutive patients were enrolled in the study. All enrolled patients had been diagnosed by chest CT, were highly suspected of having malignant pulmonary lesions, and were prepared for surgery. The details of the enrolled patients are listed in Table 1. Of the 120 highly suspected lung cancer patients enrolled, 89 were pathologically diagnosed with primary lung cancer, and 31 were diagnosed with benign nodules. The majority of patients were diagnosed with pathological stage I (p-stage I) disease (79 of 89), 2 patients had p-stage II disease, and 8 patients had p-stage III disease. A flowchart of the diagnoses of the patients enrolled in the study is shown in Figure 1.

### TBCD effectively detected CTCs in pulmonary nodule patients.

**Figure 2:**
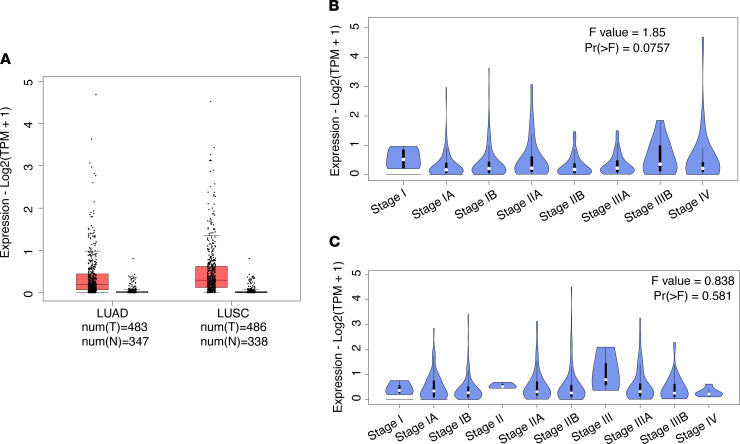
Expression of TERT in lung cancer. (**A**) TERT expression in lung carcinoma and normal samples from TCGA and the GTEx project. LUAD: tumor *n =* 483, normal *n =* 347; LUSC: tumor *n =* 486, normal *n =* 338. (**B**) Expression levels of TERT at different stages of LUAD; Pr > F = 0.0757. (**C**) Expression levels of TERT at different stages of LUSC; Pr > F = 0.581. One-way ANOVA was used for statistical analysis.

In this study, we used a live CTC detection approach based on TBCD. First, we used the online platform GEPIA2 (17) to analyze TERT expression in lung carcinoma and normal samples from The Cancer Genome Atlas (TCGA) and the Genotype-Tissue Expression (GTEx) project (http://gepia2.cancer-pku.cn/#index). The results show that TERT expression was different in lung adenocarcinoma (LUAD) samples, lung squamous cell carcinoma (LUSC) samples, and normal samples, and the expression level in LUAD or LUSC samples was higher than that in normal samples (Figure 2A). In addition, analysis of TERT at different stages of LUAD and LUSC showed that the TERT expression level was independent of stage and was higher at all stages (Figure 2, B and C). These results suggest that TBCD is feasible for detecting CTCs in patients with lung carcinoma.

**Figure 3:**
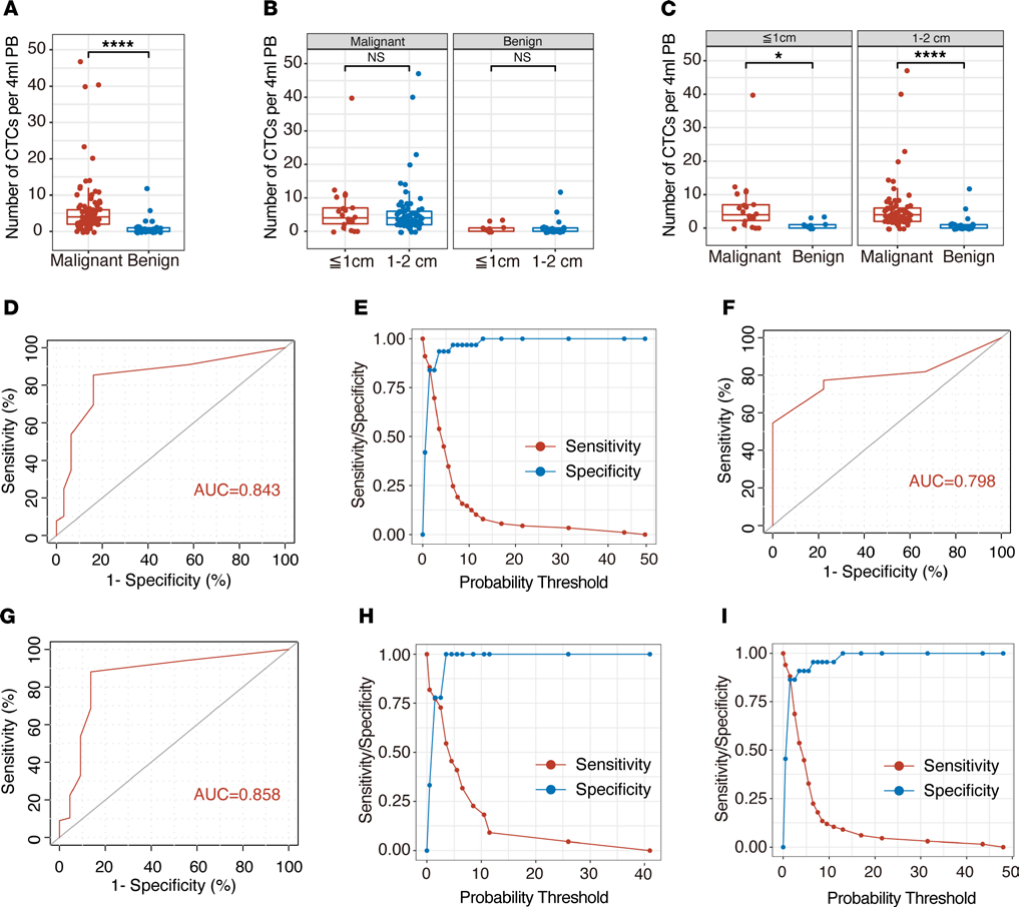
CTCs effectively detected pulmonary nodules in patients. (**A**) The average numbers of CTCs detected in the enrolled patients with benign and malignant nodules. Malignant, *n* = 89; benign, *n* = 31. Statistical analysis performed with nonparametric Mann–Whitney *U* test; ****P < 0.0001. (**B**) The average numbers of CTCs detected in the diameter groups in patients with benign and malignant nodules: malignant (≤ 1 cm, *n* = 22; 1–2 cm, *n* = 67); benign (≤ 1 cm, *n* = 9; 1–2 cm, *n* = 22; *P* = 0.88 and *P* = 0.72, respectively). Statistical analysis performed with nonparametric Mann–Whitney *U* test. (**C**) The average numbers of CTCs detected in patients with malignant or benign nodules with different diameters: ≤ 1 cm (malignant, *n* = 22; benign, *n* = 9); 1–2 cm (malignant, *n* = 67; benign, *n* = 22). Statistical analysis performed with nonparametric Mann–Whitney *U* test; *P = 0.01 and ****P < 0.0001. (**D**) ROC analysis of TBCD-CTCs in the enrolled patients. AUC = 0.843; 95% CI, 0.759–0.927. (**E**) Youden index of TBCD-CTCs in the enrolled patients. Sensitivity was 0.854 and specificity was 0.839 at the best threshold value of 1. (**F**) ROC analysis of TBCD-CTCs in patients with ≥ 1 cm pulmonary nodules. AUC = 0.798; 95% CI, 0.644–0.952. (**G**) ROC analysis of TBCD-CTCs in patients with 1–2 cm pulmonary nodules. AUC = 0.858; 95% CI, 0.753–0.963. (**H**) Youden index of TBCD-CTCs in patients with ≤ 1 cm pulmonary nodules. Sensitivity was 0.773 and specificity was 0.778 at the best threshold value of 1. (**I**) Youden index of TBCD-CTCs in patients with 1–2 cm pulmonary nodules. Sensitivity was 0.881 and specificity was 0.864 at the best threshold value of 1.

Subsequently, we performed a statistical analysis of the CTC detection results of 120 patients with pulmonary nodules. The results show that the number of CTCs in patients with malignant nodules was significantly different from that in patients with benign nodules (Figure 3A). The average number of CTCs in patients with malignant nodules was 6.05 ± 0.85 cells/4 mL PB, and the average number of CTCs in patients with benign nodules was 1.29 ± 0.43 cells/4 mL PB (*P <* 0.0001; Table 2). The details of CTC detection of enrolled patients are listed in Supplemental Table 1 (supplemental material available online with this article; https://doi.org/10.1172/jci.insight.148182DS1). Next, we analyzed the relationship between pulmonary nodule size and the number of CTCs. We divided the patients into the ≤ 1 cm nodule group and the 1–2 cm nodule group according to the preoperative CT detection results of the size of the pulmonary nodules. The results show that there was no statistically significant difference in the number of CTCs between patients with ≤ 1 cm nodules (6.27 ± 1.80 cells/4 mL PB for malignant and 1.11 ± 0.39 cells/4 mL PB for benign) and patients with 1–2 cm nodules (5.97 ± 0.96 cells/4 mL PB for malignant and 1.36 ± 0.58 cells/4 mL PB for benign), regardless of whether the patients had malignant or benign pulmonary nodules (*P =* 0.88 for malignant and *P =* 0.72 for benign; Figure 3B). We divided the patients into the ≤ 1 cm nodule group (31 of 120) and the 1–2 cm nodule group (89 of 120) according to the size of the pulmonary nodules. In the above 2 groups, there were significant differences in the number of CTC tests in patients with benign and malignant pulmonary nodules (*P =* 0.01 and *P <* 0.0001, respectively; Figure 3C). As shown in Figure 3D, receiver operating characteristic (ROC) curve analysis showed that the AUC was 0.843 (95% CI, 0.759–0.927). When the threshold value was 1, the sensitivity and specificity of detection were 0.854 and 0.839, respectively (Figure 3E). Moreover, in the analysis of groups by nodule size, TBCD had excellent detection efficiency (Figure 3, F and G). The AUCs were 0.798 (95% CI, 0.644–0.952) and 0.858 (95% CI, 0.753–0.963) in the ≤ 1 cm nodule group and the 1–2 cm nodule group, respectively. When the threshold value was 1, the sensitivity and specificity of the ≤ 1 cm nodule group were 0.773 and 0.778, and those of the 1–2 cm nodule group were 0.881 and 0.864, respectively (Figure 3, H and I). The details are listed in Supplemental Table 2. The results show that CTC detection could be defined as a positive result when the number of CTCs was greater than or equal to 2. Based on this threshold, this method had a high differentiating efficiency for benign and malignant pulmonary nodules.

### FlowSight and FISH confirmed the CTCs detected by TBCD.

**Figure 4:**
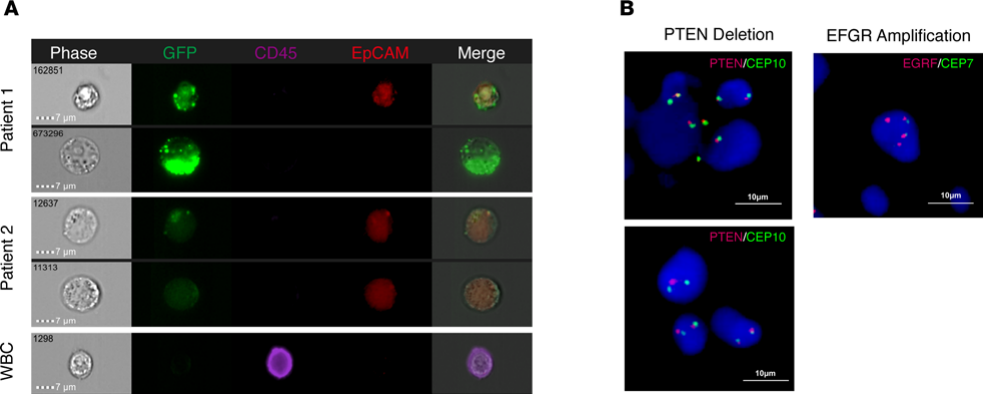
CTC confirmation via FISH and FlowSight. (**A**) CD45-eFluor405 (purple), hTERTp-GFP (green), EpCAM (red), and bright-field digital images are shown for CTCs and WBCs. CTCs derived from malignant nodules are marked by GFP with or without the EpCAM marker. CTCs were defined as CD45^–^/GFP^+^/EpCAM^+^ or CD45^–^/GFP^+^/EpCAM^–^ cells. WBCs were only marked with CD45. Scale bar: 7 μm. (**B**) Dual-color FISH results of the PTEN (10q23.3) heterozygous deletion and EGFR (7p11.2) amplification in CTCs from patients with malignant pulmonary nodules. Scale bar: 10 μm.

To verify that the CTCs were derived from the subsection junction, FlowSight imaging and FISH experiments were performed. The detection method of CTCs was modified, EpCAM antibodies were added in the detection stage, and finally, the fluorescence tracer of CTCs was displayed through the FlowSight system. As shown in Figure 4A, CTCs were slightly larger than WBCs, and GFP expression under control of the human TERT (hTERT) promoter was observed with or without EpCAM expression (CD45^–^GFP^+^EpCAM^+^ or CD45^–^GFP^+^EpCAM^–^). Additionally, WBCs only expressed CD45 (CD45^+^GFP^–^EpCAM^–^). We found that there was a heterogeneity in CTCs among different patients or in the same patient. In patient 1, CD45^–^GFP^+^EpCAM^+^ and CD45^–^GFP^+^EpCAM^–^ CTCs could be found at the same time. However, in patient 2, we observed the presence of only CD45^–^GFP^+^EpCAM^+^ CTCs. We used FISH to detect PTEN deletion or EGFR amplification in patients with CTCs. In patients with positive CTC test results, we found a typical signal configuration with heterozygous deletion of the PTEN gene (Figure 4B). The detected EGFR amplification was also confirmed by FISH (Figure 4B). These results suggest that the CTCs detected were derived from malignant nodules.

### TBCD CTCs were independent of serum tumor markers in malignant nodule patients.

**Figure 5:**
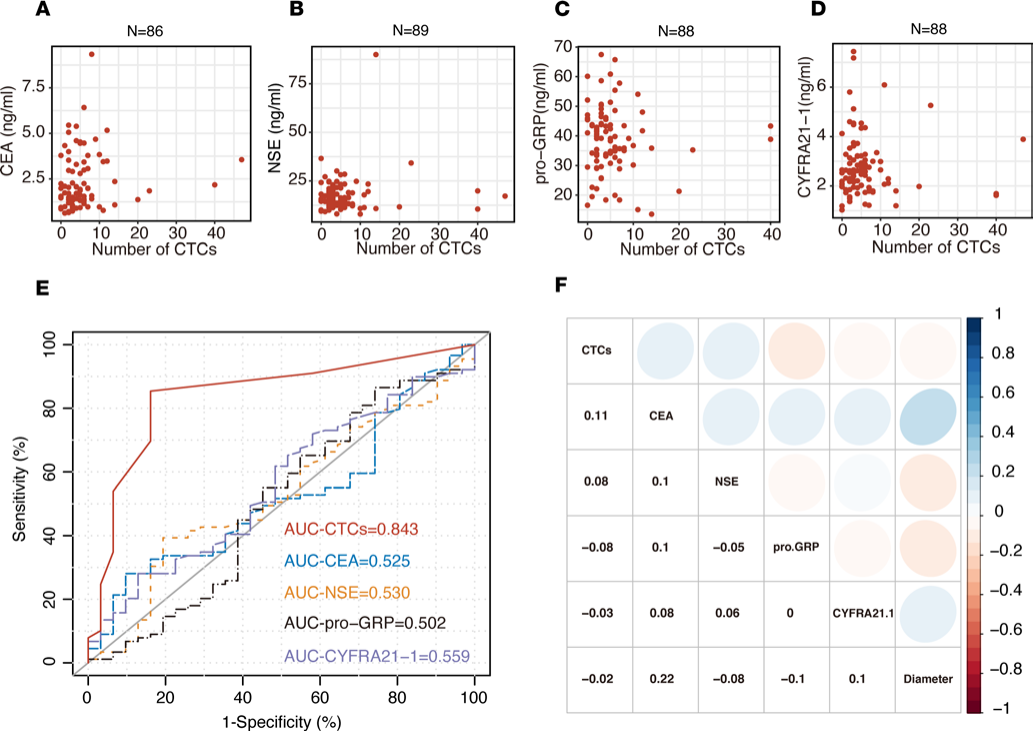
Correlation between CTCs and tumor biomarkers. (**A**) There was no association between the levels of CTCs and CEA in patients with malignant pulmonary nodules (*n =* 86; Spearman’s correlation analysis). (**B**) There was no association between the levels of CTCs and NSE in patients with malignant pulmonary nodules (*n =* 89; Spearman’s correlation analysis). (**C**) There was no association between the levels of CTCs and pro-GRP in patients with malignant pulmonary nodules (*n =* 88; Spearman’s correlation analysis). (**D**) There was no association between the levels of CTCs and CYFRA21-1 in patients with malignant pulmonary nodules (*n =* 88; Spearman’s correlation analysis). (**E**) ROC curve analysis of the numbers of CTCs and the levels of CEA, NSE, pro-GRP, and CYFRA21-1 in the enrolled patients with pulmonary nodules. (**F**) Spearman’s correlations between CTCs and tumor biomarkers and nodule diameters in patients with malignant pulmonary nodules, displayed as heatmaps. Positive correlations are shown in blue-based colors, while negative correlations (anticorrelations) are shown in red-based colors.

Subsequently, we analyzed the correlation between the number of CTCs and serum tumor markers (CEA, NSE, pro-GRP, and CYFRA21-1) in malignant nodule patients. Patients with tumor marker recordings 5 times higher than the detection threshold (all of these patients were positive for CTCs) were excluded because severely deviated sample information would lead to a false linear relationship in the correlation analysis. The correlation results are shown in Figure 5, A–D. As shown in Figure 5E, by using logistic regression, we found that the number of CTCs was not correlated with the expression of serum tumor markers and was an independent factor (Supplemental Table 3). Compared with the ROC curve analysis of serum tumor markers, TBCD was superior in determining malignant and benign nodules. The AUC of our approach was 0.843 (95% CI, 0.759–0.927), and the AUCs for the serum CEA level, NSE level, pro-GRP level, and CYFRA21-1 level were 0.524 (95% CI, 0.412–0.637), 0.530 (95% CI, 0.414–0.646), 0.502 (95% CI, 0.375–0.629), and 0.559 (95% CI, 0.445–0.673), respectively (Figure 5F). TBCD was better than all other serum models and was significantly different (*P <* 0.0001, *P <* 0.0001, *P <* 0.0001, and *P =* 0.0001, respectively; Supplemental Table 4). The results also show that the number of CTCs detected by TBCD was independent of the diameter of pulmonary nodules. These results indicate that CTCs are useful markers of pulmonary nodule malignancy and that TBCD can effectively discriminate benign and malignant pulmonary nodules.

### TBCD could effectively assist CT in pulmonary nodule diagnosis.

**Figure 6:**
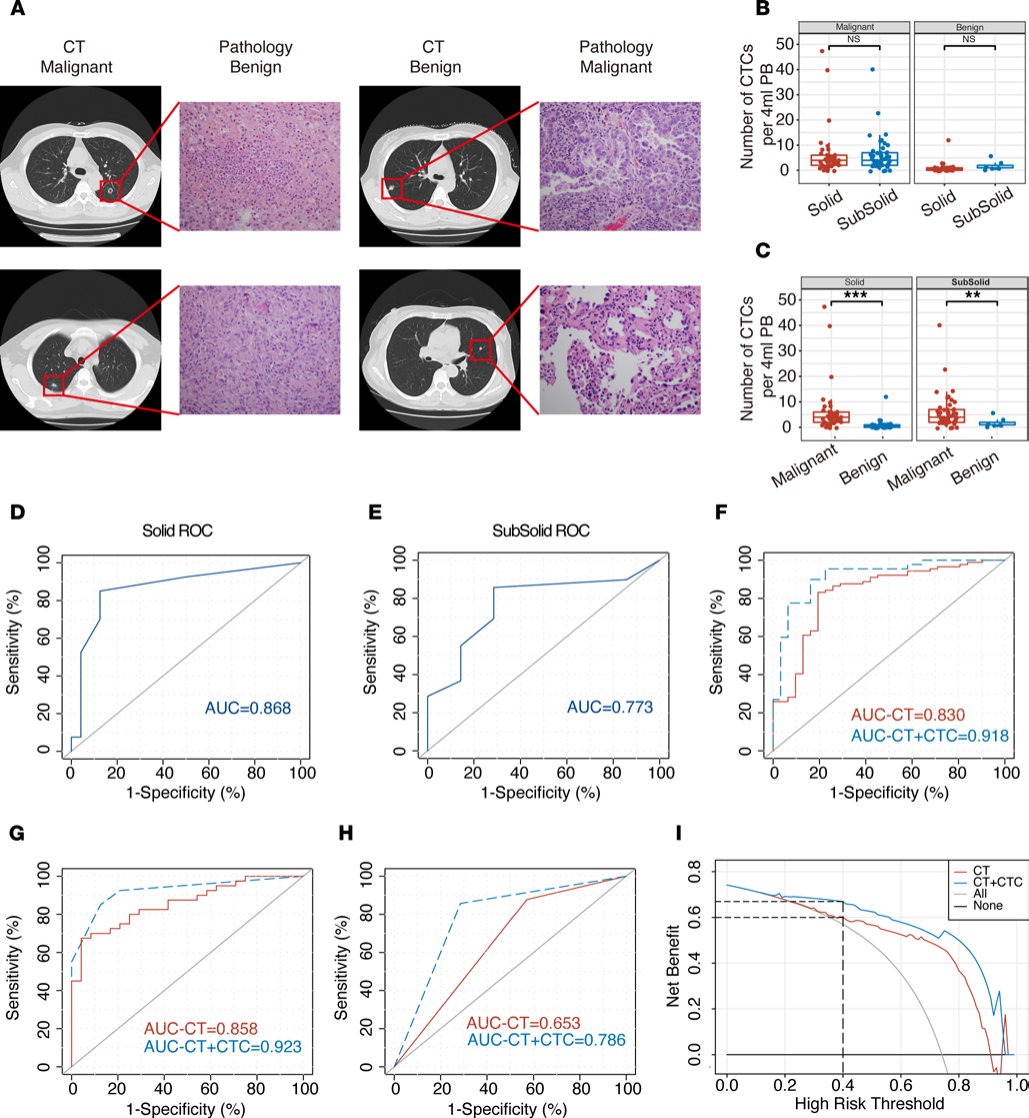
Comparison of TBCD with CT in the enrolled patient cohort. (**A**) The results of a typical CT examination were inconsistent with those of postoperative pathology. Total original magnification, ×200. (**B**) The average numbers of CTCs detected in patients with malignant or benign nodules with different densities. Malignant (solid, *n =* 40; subsolid, *n =* 49); benign (solid, *n =* 24; subsolid, *n =* 7). Statistical analysis performed with nonparametric Mann–Whitney *U* test; *P =* 0.77 and *P =* 0.44, respectively. (**C**) The average numbers of CTCs detected in patients with different types of malignant or benign nodules. Solid (malignant, *n =* 40; benign, *n =* 24); subsolid (malignant, *n =* 49; benign, *n =* 7); *P =* 0.0018 and *P =* 0.003, respectively. Statistical analysis performed with nonparametric Mann–Whitney *U* test; **P < 0.01; ***P < 0.001. (**D**) ROC curve analysis of the numbers of CTCs in patients with solid nodules. AUC = 0.868; 95% CI, 0.769–0.967. (**E**) ROC curve analysis of the numbers of CTCs in patients with subsolid nodules. AUC = 0.773; 95% CI, 0.599–0.946. (**F**) ROC curve analysis of CT and CT combined with CTCs in the enrolled patients with pulmonary nodules. AUC-CT = 0.830; 95% CI, 0.741–0.919. AUC-CT + CTC = 0.918; 95% CI, 0.858–0.977. (**G**) ROC curve analysis of CT and CT combined with CTCs in patients with solid nodules. AUC-CT = 0.858; 95% CI, 0.770–0.947. AUC-CT + CTC = 0.923; 95% CI, 0.857–0.989. (**H**) ROC curve analysis of CT and CT combined with CTCs in patients with subsolid nodules. AUC-CT = 0.653; 95% CI, 0.410–0.896. AUC-CT + CTC=0.786; 95% CI, 0.580–0.991. (**I**) Decision curve analysis for pulmonary nodule diagnosis. Red line, CT diagnosis only; blue line, CT combined with CTC diagnosis.

Although the development of CT technology has made the detection of pulmonary nodules easier, approximately 20% of the pulmonary nodules resected in clinical surgery are still benign lesions, and the qualitative diagnosis of pulmonary nodules is still very difficult. As shown in Figure 6A, among the patients enrolled in this study, postoperative pathology showed benign nodules in patients with highly suspicious CT results and showed malignant tumors in patients with benign nodules in the same way. The positive rate of CTCs (CTC > 1 defined as positive) was 85.4% for malignant nodules and 16.1% for benign nodules (Supplemental Figure 1). Therefore, we further compared the correlation between TBCD and CT. According to the CT results, we divided the 120 enrolled patients into the solid nodule group (64 of 120) and the subsolid nodule group (56 of 120). The results show that there was no significant difference in the average number of CTCs detected in patients with solid nodules and in those with subsolid nodules (6.325 ± 1.485 versus 5.816 ± 0.955, *P =* 0.77) in the malignant nodule group (Figure 6B and Table 2). In the benign nodule group, the average number of CTCs detected was also not significantly different (1.125 ± 0.505 versus 1.875 ± 0.769, *P =* 0.44; Figure 6B and Table 2). By comparing malignant and benign nodules, the average number of CTCs detected was significantly different in both the solid nodule and subsolid nodule groups (*P =* 0.0018 and *P =* 0.003, respectively; Figure 6C and Table 2). ROC curve analysis showed that the AUC of the solid nodule group was 0.868 (95% CI, 0.769–0.967), while the AUC of the subsolid nodule group was 0.773 (95% CI, 0.599–0.946), indicating that TBCD was effective in differentiating benign from malignant nodules in different groups (Figure 6, D and E). To predict whether a pulmonary nodule is malignant, chest CT features including size, density, growth, and specific morphology features can be used. We obtained parameters for imaging (including spicular sign, lobulation, pleural indentation, vacuole sign, aerial bronchogram, vessel convergence, mean CT value, and node diameter), and logistic regression was used for model fitting. Then, ROC curve analysis was conducted for the independent CT evaluation and the CT combined with CTC evaluation of the enrolled patients. The results show that the AUC of CT alone was 0.830 (95% CI, 0.741–0.919), and the AUC of CT combined with CTCs was 0.918 (95% CI, 0.858–0.977), suggesting that CT combined with CTCs was a better approach for the diagnosis of pulmonary nodules (*P =* 0.040; Figure 6F and Table 3). Subsequently, in the solid nodule group and the subsolid nodule group, the AUCs of CT alone were 0.858 (95% CI, 0.770–0.947) and 0.653 (95% CI, 0.410–0.896), respectively, and the AUCs of CT combined with CTCs were 0.923 (95% CI, 0.857–0.989) and 0.786 (95% CI, 0.580–0.991), respectively, with no significant difference between the 2 methods (*P =* 0.16 and *P =* 0.37; Figure 6, G and H). Finally, decision curve analysis (DCA) was performed to compare the clinical effects of CT alone and CT combined with CTCs (Figure 6I). Interestingly, although CT evaluation showed that patients could benefit clinically when the threshold was 0.4, CT combined with CTC evaluation provided clinical benefit to patients at a lower threshold. In addition, within the threshold range of more than 0.4, the clinical benefit of patients evaluated by CT combined with CTCs was significantly higher than that evaluated by CT alone. These results show that TBCD is an effective method to assist CT in the determination of benign and malignant nodules in the lung and can significantly improve the accuracy of CT in the determination of benign and malignant nodules.

## Discussion

In this study, we used the TBCD assay to distinguish benign from malignant nodules in patients with nodules < 2 cm in size and compared this method with the pathological diagnosis as the gold standard. Our results show that the number of CTCs detected based on TBCD was not correlated with serum tumor markers. Thus, TBCD could be used as an independent biological indicator for the determination of benign and malignant nodules and showed a significantly better differentiation ability than serum tumor markers. TBCD improved the detection capability in both the ≤ 1 cm and 1–2 cm pulmonary nodule groups based on the optimal threshold of 1 recommended by the ROC curve (patients with 2 or more CTCs were considered positive). In addition, logistic regression analysis with multiple CT test indexes showed that the CTC test as an auxiliary CT test could significantly improve the ability to distinguish benign from malignant nodules in lung nodules smaller than 2 cm.

Lung cancer is a malignant tumor with the highest morbidity and mortality in the world, and the most effective control methods are early screening, early diagnosis, and early treatment. As the initial stage of pulmonary disease progression, pulmonary nodules may be early lesions of lung cancer. The correct diagnosis of pulmonary nodules and the determination of benign and malignant nodules are of great significance for the corresponding clinical treatment (18).

With the emergence of liquid biopsy techniques represented by CTCs and ctDNA, an increasing number of studies have focused on the relationship between CTCs and the prediction and prognostic assessment of lung cancer (10, 11, 19, 20). At present, the methods of CTC capture and enrichment are basically divided into 2 categories (21). The first is based on physical methods. Such an approach can be used to screen CTCs by differences in cell size or density. The other is the capture of CTCs by immune binding based on cell surface markers. Most of the studies reported so far have adopted the combination of the above 2 approaches, such as positive and negative screening schemes through cell size combined with surface markers. In addition, further identification methods, such as reverse transcription PCR (RT-PCR) and immunofluorescence labeling, are often used after the capture of enriched CTCs (22, 23). These different approaches have their own advantages and disadvantages. The enrichment and separation methods based on physical characteristics tend to be biased, resulting in a decrease in detection sensitivity and specificity. However, the detection method based on surface markers is often highly sensitive but produces false-positive signals. The root cause is that there is no recognized accurate tumor surface marker at this stage. A CellSearch-based primary lung cancer diagnostic study reported that, although CTC counts were significantly different in lung cancer patients and nonmalignant patients, ROC curve analysis showed that the sensitivity and specificity of the test were lower than those of the serum marker CEA (30.4 versus 45.6 for sensitivity, 88.0 versus 92.0 for specificity; ref. 11). CellSearch-based CTCs are more suitable as biomarkers for predicting primary metastasis than for early identification. A recent study has also shown that the enrichment and separation of CTCs using the isolation by size of epithelial tumor cell technique was not suitable for lung cancer screening (24). Some studies on the diagnosis of lung cancer based on folic acid receptors have shown that the method has a certain determination efficacy in detection, with a sensitivity between 72.0 and 76.4 and a specificity between 73.8 and 84.1 (10, 25, 26). However, its detection ability is often related to tumor size and stage, and the detection efficiency in p-stage I patients is lower than that in other stage patients (sensitivity is 67.2; ref. 27). Some studies have also shown the clinical application potential of ctDNA in benign and malignant screening for small nodules of lung cancer. Chen et al. assessed the diagnostic accuracy of plasma samples for early lung cancer based on promoter methylation in 8 lung cancer-specific genes (CDO1, TAC1, SOX17, HOXA7, HOXA9, GATA4, GATA5, and PAX5) in plasma samples. Among these genes, a combination of CDO1, SOX17, and HOXA7 could achieve a sensitivity and specificity of 90% and 71%, respectively (27). Liang et al. investigated protocols of 10,560 prospective, observational, and multicenter clinical trials (28). They conducted a multicenter clinical study of methods for detecting ctDNA methylation in plasma samples with a sensitivity and specificity of 82.5% and 83.3%, respectively. Exosome-based benign and malignant screening for small nodules of lung cancer has rarely been reported because of its late start. Kuang et al. first reported the role of plasma exosomal fibrinogen β chain (FGB) and fibrinogen γ chain (FGG) in the prediction of benign or malignant pulmonary nodules (29). Since ctDNA and exosomes exist in plasma, the combined application of CTCs with ctDNA or exosomes to the same sample may be the way to further improve diagnostic accuracy between benign and malignant pulmonary nodules.

In our study, a CTC enrichment and separation approach based on TERT was adopted. The approach was achieved by using TERT activity, which maintains the unlimited replication potential of tumor cells, as a positive screening marker combined with the negative screening of CD45 antibody markers. The results show that, in LUAD, the expression level of TERT in tumor samples was significantly different from that in normal samples, and the expression level of TERT in tumor samples was not correlated with stage. These results suggest that TBCD is feasible for differentiating malignant and benign lung cancer patients and that the ability to differentiate between patients at different stages should be uniform. The detection results of the 120 enrolled patients with pulmonary nodules < 2 cm in size showed that, with a threshold of 1, the overall detection AUC was 84.3, and the detection sensitivity and specificity reached 85.4 and 83.9, respectively. The enrolled patients were further grouped. In the 1–2 cm nodule group, the detection AUC of this method was 85.8, and the sensitivity and specificity were 88.0 and 86.4, respectively. In the ≤ 1 cm nodule group, the AUC was 79.8, and the sensitivity and specificity were 77.3 and 77.8, respectively. The results show that TBCD exhibited robust efficacy in the screening of benign and malignant pulmonary nodules in diagnostic experiments using pathology as the gold standard.

For some patients with pulmonary nodules, conventional diagnostic methods such as fine-needle aspiration and transbronchial biopsy are often only able to obtain small tumor specimens, as it is difficult to obtain samples with these methods (30). Therefore, in PB, the assessment of tumor markers is of clinical value for the determination of benign and malignant tumors (31). Because of the low concentration of clinically common tumor markers in the PB of patients with pulmonary small nodules, it is difficult to use them as independent biomarkers for diagnosis (31). Some studies have shown that the combination of multiple tumor markers in serum can distinguish cancer patients from healthy people (32, 33). Although research on tumor markers for lung cancer has made great progress, there is still no breakthrough in tumor markers with high specificity and sensitivity, especially for early- or precancerous-stage lung cancer, and no suitable tumor markers can be used for diagnosis and application. In our study, we performed ROC curve analysis of serum tumor markers (CEA, NSE, pro-GRP, and CYFRA21-1) in the 120 enrolled patients with pulmonary nodules. The results show that there was little difference in the individual detection efficacy between these markers, and the AUC values were all less than 0.6, suggesting that they were not suitable as biomarkers for the diagnosis of lung cancer. In some studies, the CEA expression level was shown to have a certain application value in the determination of pulmonary nodule benignity and malignancy (11, 33, 34), while in this study, it was not shown to have an application value. This may be because serum CEA expression levels in patients with pulmonary nodules < 2 cm tend to be within a normal detection threshold range. Compared with serum markers, TBCD performed significantly better than the above 4 serum tumor markers (all *P* < 0.001). With a threshold of 1, the sensitivity and specificity reached 0.854 and 0.839, respectively. In addition, Pearson’s correlation coefficient showed that CTCs had no correlation with the above serum markers and could be used as independent biomarkers to determine benign and malignant lung cancer tumors. Compared with serum tumor markers, TBCD alone or in combination with other markers may be a more effective technical approach for differentiating benign from malignant pulmonary nodules.

At present, early-stage lung cancers are mainly diagnosed as isolated lung nodules by chest CT and are divided into solid and subsolid nodules based on their respective differentiation components — the latter containing pure ground-glass nodules (pGGNs) and part-solid nodules (PSNs). Previous studies have shown that chest CT plays an important role in the differential diagnosis of benign and malignant pulmonary nodules with specific morphological features or CT values (e.g., radiology) and even some CT characteristics, such as components, pathological subtypes, gene mutations, and prognostic correlation (35, 36). With the popularization and application of LDCT in early lung cancer screening, a large number of pulmonary nodules have been found, and the main problem is overdiagnosis and overtreatment, with studies showing false-positive rates of lung cancer exceeding 18.5% (37). This was due to CT radiologic features of benign and malignant pulmonary nodules overlapping, making them difficult to distinguish (Figure 6A). In theory, CTC detection by a noninvasive liquid biopsy approach can be used to avoid the interference of the complex signs of CT and serve as an adjunctive approach to help CT differentiate and diagnose pulmonary nodules. In our study, we not only evaluated the efficacy of CT alone in the enrolled patients, but we also conducted a retrospective evaluation of the combination of TBCD with CT. By regression fitting of imaging parameters (including spicular sign, lobulation, pleural indentation, vacuole sign, aerial bronchogram, vessel convergence, mean CT value, and tumor diameter), the sensitivity and specificity of CT as a single diagnostic method in this study reached 83.1 and 80.6 in all patients, respectively. The patient population was divided into a solid nodule group and a subsolid nodule group, and CT alone showed high specificity and low sensitivity (67.5 for sensitivity and 95.8 for specificity) in the solid nodule group or high sensitivity and low specificity (87.8 for sensitivity and 42.6 for specificity) in the subsolid nodule group. The diagnostic efficiency of TBCD combined with CT in the diagnosis of pulmonary nodules was better than that of CT alone (*P =* 0.039), and the sensitivity and specificity improved to 89.9 and 83.9, respectively. The diagnosis in different subgroups showed that TBCD combined with CT could achieve a more balanced detection sensitivity and specificity. Therefore, these results indicate that TBCD can be an excellent auxiliary technique to improve the accuracy of evaluating the nature of pulmonary nodules by CT, as well as to reduce the anxiety, further costly evaluation, cumulative radiation hazard, and pain caused by invasive examination.

To more comprehensively evaluate the diagnostic value of a test, it is necessary to consider all possible diagnostic thresholds. ROC curve analysis is widely used in the performance evaluation of medical diagnostic tests (38). When the AUC is 0.7–0.9, the diagnostic accuracy is moderate, and when the AUC is above 0.9, the diagnostic accuracy is high. DCA is also used in studies to evaluate the diagnostic value of diagnostic tests (39). To ensure the repeatability and accuracy of TBCD, we also conducted test validation using 3 batches. The simulated samples were tested for accuracy, precision, and specificity. The linear regression coefficients of different batches were all higher than 0.99 (data not shown). The coefficient of variation between test batches and batch precision ranged from 5% to 8% (data not shown). These results demonstrate that TCBD is an accurate and reproducible CTC assay.

In this study, there were some patients with false-positive or false-negative results. The false negatives may have been due to the principle of detection and the periodicity of CTCs, which should be further optimized in subsequent studies. Among the patients with false-positive results, 3 patients had atypical adenomatoid hyperplasia and should be followed up with to assess their true status. CD45^–^GFP^+^ CTCs could also be detected in patients with benign lesions, which might have been due to the following reasons. (a) Limited specificity of the method. In the peripheral circulation, there are a large number of juvenile WBCs, which could cause false-positive results. (b) These patients may have had undetected tumor lesions that released the CTCs detected, resulting in positive results. Therefore, patients with benign lesions with positive results can be further excluded based on multiple tests and a follow-up.

This study has the following shortcomings and improvements. (a) As an exploratory study, the sample size of enrolled patients was small. It is necessary to further expand the sample size and carry out multicenter verification. (b) The patients enrolled in this study were highly suspected of having lung cancer by CT diagnosis, and TBCD can also be further verified in LDCT to further explore the ability of TBCD combined with LDCT in lung cancer screening. (c) With the development of artificial intelligence (AI) diagnostic technology, the combination of AI diagnostic technology with CT and TBCD may achieve a better diagnostic performance.

In conclusion, TBCD can improve the diagnosis of pulmonary nodules and can be used as a robust auxiliary diagnostic scheme for CT diagnosis.

## Methods

### Patients and sample collection.

In total, 120 patients newly diagnosed by CT were recruited, and the clinical information of the included patients was provided by the Department of Thoracic Surgery, Beijing Chest Hospital. Blood (4 mL) was collected from eligible patients with K_2_E (EDTA) tubes, kept at 4°C, and transported to the laboratory within 2 hours.

### Blood sampling treatment and CTC identification.

The 4 mL blood samples were centrifuged at 500*g* for 5 minutes at room temperature, and the plasma was discarded. Then, RBC lysis buffer (NH_4_Cl, 0.15M; EDTA, 0.1 mM; KHCO_3_, 10 mM; pH 7.2) was added to the samples. The samples were centrifuged at 500*g* for 5 minutes at room temperature, the supernatant was discarded, and 5 mL of 1× PBS was added to wash the cells. The samples were centrifuged again at 500*g* for 5 minutes at room temperature, and 2 mL of serum-free medium was used to resuspend and seed all cells from a 4 mL blood sample. CTCs were detected using reagent (oHSV1-hTERTp-GFP) as previously described (13). Cells transduced with oHSV1-hTERTp-GFP were incubated in a humidified atmosphere of 5% CO_2_ at 37°C for 24 hours. The transduced cells were harvested and stained with an APC-CD45 antibody (HI30, Invitrogen). CD45^−^GFP^+^ cells were recorded as TBCD-CTCs for TBCD (Supplemental Figure 2).

### Identification of CTCs using FlowSight and FISH.

The samples were subjected to a standard CTC identification process. For flow imaging, all cells from 4 mL blood samples were collected and incubated with an eFluor405 anti-CD45 antibody (HI30, BioLegend) and APC anti-EpCAM antibody (CO17-1A, BioLegend). The incubation time was 30 minutes at room temperature. After washing, CTCs were detected by the ImageStreamX Mark II system (FlowSight, Amnis). CD45^–^GFP^+^EpCAM^+^ cells in blood samples were considered CTC^+^ cells (Supplemental Figure 3).

For FISH, MACS magnetic human CD45 microbeads (Miltenyi Biotec) were used to isolate CD45^–^ cells. After fixation and sectioning, the cells were labeled with a dual-color FISH probe set consisting of a Spectrum Red–labeled PTEN gene probe (CELNOVTE) in the chromosome 10q23.3 region and a Spectrum Green–labeled CEP10 gene probe (CELNOVTE FISH system). DAPI was used to stain the nuclei. The predominantly red and green signal numbers were recorded for each FISH probe. Heterozygous deletion of PTEN was defined as the presence of fewer PTEN signals compared with the centromere 10 probe signal. The cells were also labeled with a dual-color FISH probe set consisting of a Spectrum Red–labeled EGFR gene probe (CELNOVTE) in the chromosome 7p11.2 region and a Spectrum Green–labeled CEP7 gene probe (CELNOVTE FISH system). DAPI was used to stain the nuclei. The numbers of predominantly red and green signals were recorded for each FISH probe. A positive EGFR/CEP7 ratio ≥ 2.0 indicated EGFR gene amplification in a sample.

### Statistics.

Statistical analysis of the data was carried out with standard software (IBM SPSS Statistics 26.0 and Prism 8, USA). The nonparametric Mann–Whitney *U* test was used to test 2 patient groups (categorical and continuous data). Pearson’s χ^2^ test was used to test the expected frequencies and the observed frequencies in 2 categories of a contingency table. ROC curves were constructed based on the diagnostic efficiency of tumor biomarkers and CTCs, and the AUC represented the diagnostic performance. Logistic regression was used to calculate the predictive probability for the combined methods of CT and CTCs. Clinical usefulness was evaluated by DCA. Data are expressed as the mean ± SEM. All *P* values were 2 sided, with *P* < 0.05 considered statistically significant.

### Study approval.

This study was approved by the ethics committee of the Beijing Chest Hospital, Capital Medical University (KY-2018-004). Written informed consent was obtained from the enrolled patients.

## Author contributions

WZ, XD, ZCY, and CZ performed the experiments. All authors participated in designing various parts of the study and in discussion and interpretation of the results. XD, ZZ, and ZL collected the samples and clinical information. WZ, XD, and ZY performed the data analysis. WZ and XD wrote the manuscript with input from all authors. WZ, SC, and KZ supervised the study.

## Supplementary Material

Supplemental data

ICMJE disclosure forms

## Figures and Tables

**Table 3:**
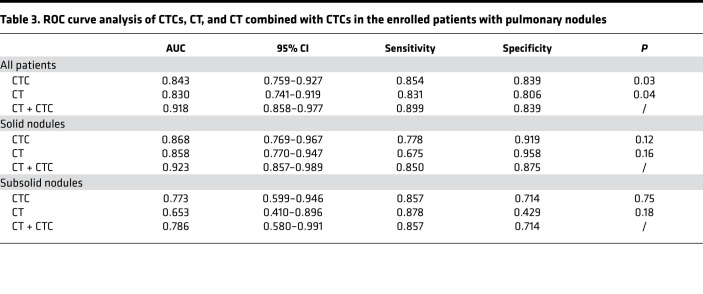
ROC curve analysis of CTCs, CT, and CT combined with CTCs in the enrolled patients with pulmonary nodules

**Table 2:**
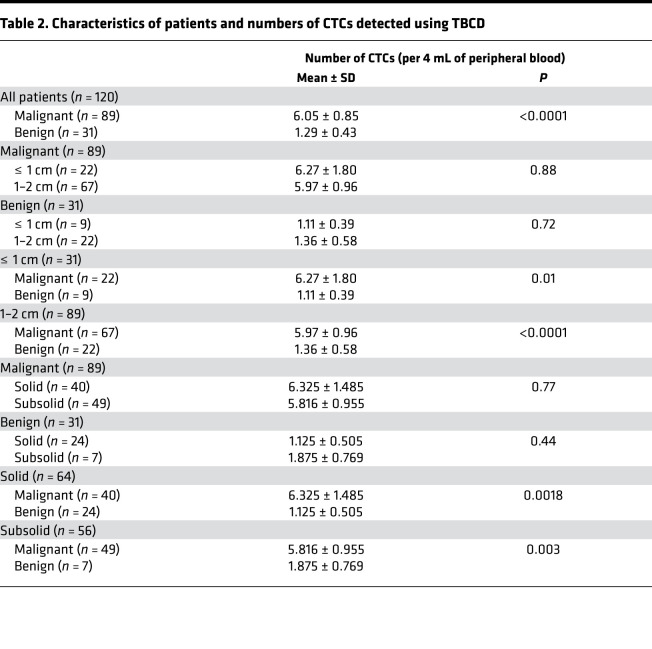
Characteristics of patients and numbers of CTCs detected using TBCD

**Table 1:**
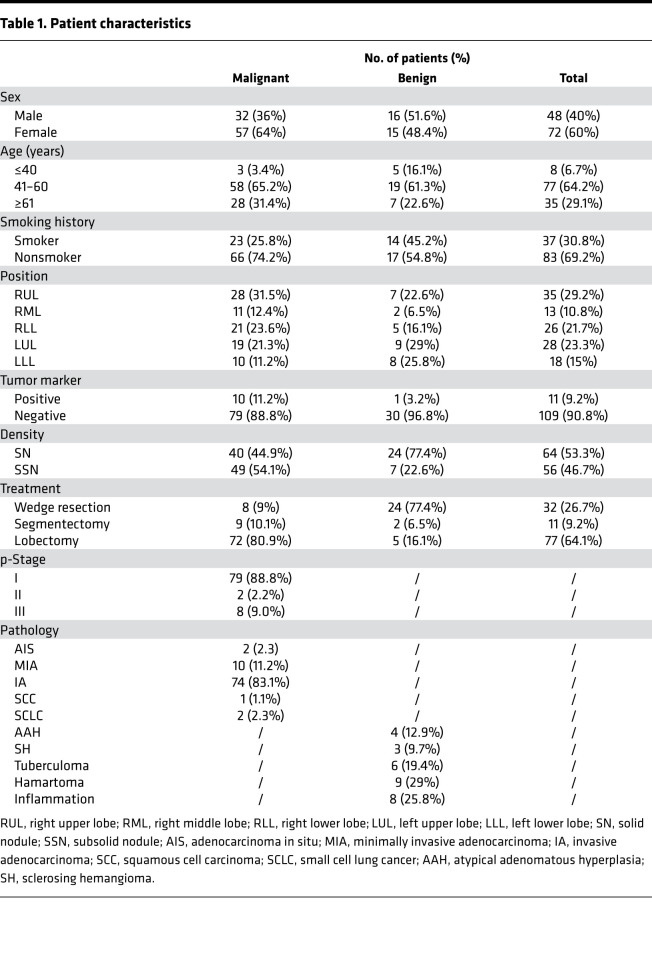
Patient characteristics

## Version 1 04/27/2021

In-Press Preview

## Version 2 06/08/2021

Electronic publication
