# Supplementary material for: Combination of CT and telomerase+ circulating tumor cells improves diagnosis of small pulmonary nodules
Source: JCI Insight. 2021 Jun 8;6(11):e148182. doi: 10.1172/jci.insight.148182 (PMC8262359; doi:10.1172/jci.insight.148182)
Supplement: Supplemental data [file jciinsight-6-148182-s089.pdf]

Supplymentary Figure and Legend

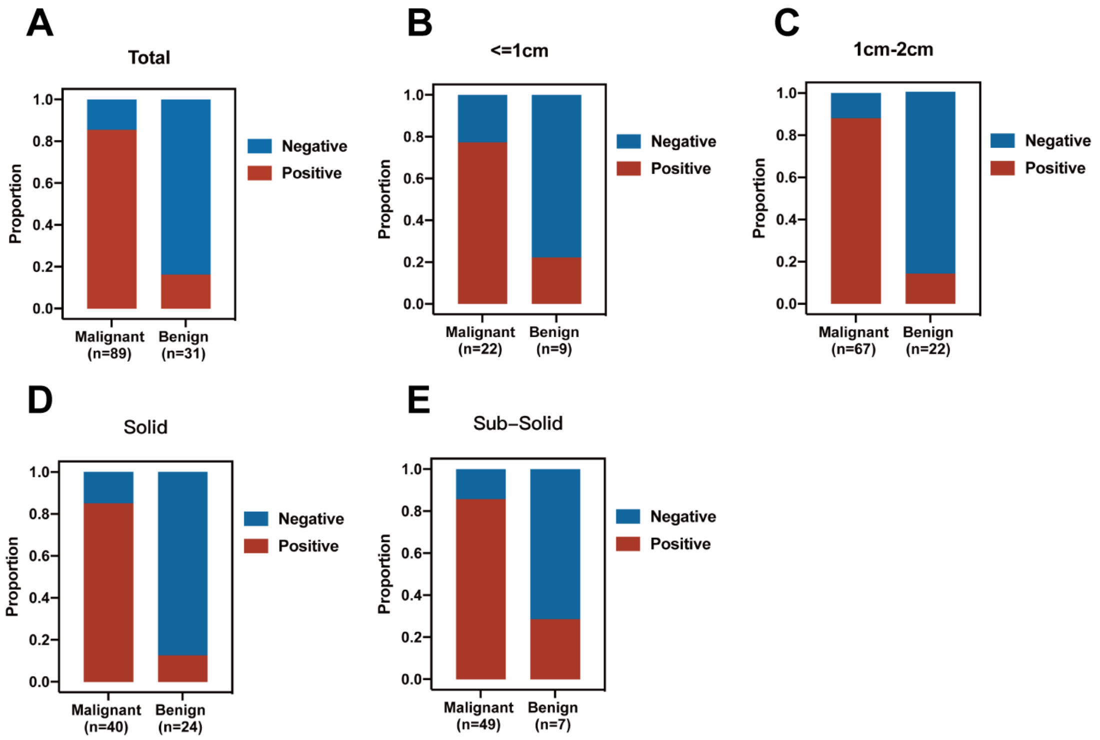

Supplymentary Figure 1. Positive rate of CTC sample test.

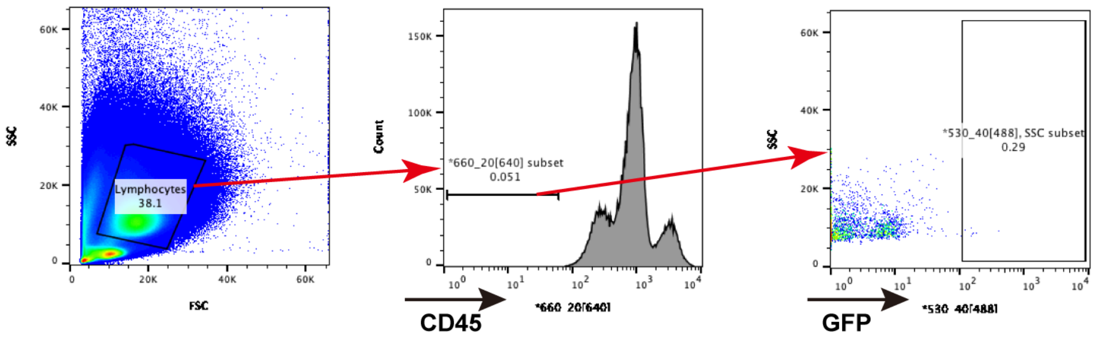

Supplymentary Figure 2. Flow analysis of CTC sample detection.

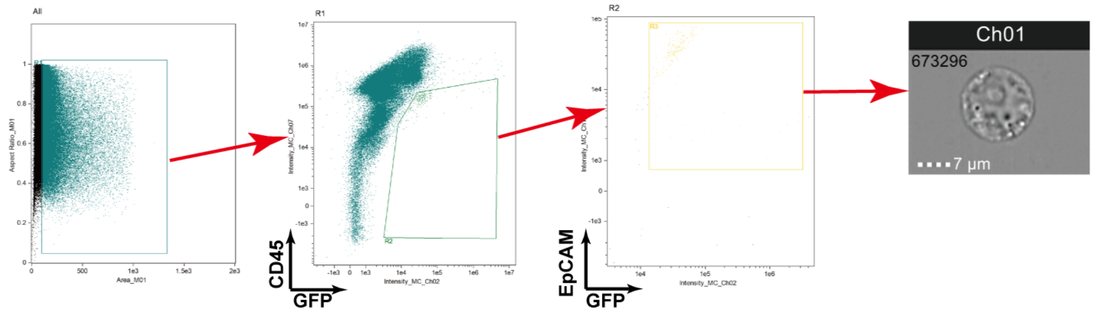

Supplymentary Figure 3. FlowSight analysis of CTC sample detection.

Supplyment Table

Supplementary Table 1. The number of CTCs for each enrolled patient.

NO	CTC number per 4ml PB	CT (diameter mm)	Density	Type
1	1	0.9	Solid nodule	Malignant
2	4	2	Subsolid nodule	Malignant
3	11	0.8	Subsolid nodule	Malignant
4	7	0.6	Subsolid nodule	Malignant
5	20	1.1	Solid nodule	Malignant
6	6	1.7	Subsolid nodule	Malignant
7	14	1.3	Subsolid nodule	Malignant
8	6	1.2	Solid nodule	Malignant
9	10	0.6	Subsolid nodule	Malignant
10	1	2	Subsolid nodule	Malignant
11	8	2	Solid nodule	Malignant
12	2	2	Solid nodule	Malignant
13	3	1	Solid nodule	Malignant
14	4	2	Solid nodule	Malignant
15	8	1.5	Subsolid nodule	Malignant
16	4	1.9	Solid nodule	Malignant
17	6	0.6	Subsolid nodule	Malignant

18	5	1.6	Subsolid nodule	Malignant
19	2	1.5	Subsolid nodule	Malignant
20	5	1	Solid nodule	Malignant
21	3	2	Solid nodule	Malignant
22	5	1.1	Solid nodule	Malignant
23	2	1.9	Subsolid nodule	Malignant
24	6	1.8	Subsolid nodule	Malignant
25	3	1	Subsolid nodule	Malignant
26	3	0.8	Subsolid nodule	Malignant
27	5	1.5	Subsolid nodule	Malignant
28	3	1.3	Subsolid nodule	Malignant
29	23	1.3	Subsolid nodule	Malignant
30	1	1.2	Solid nodule	Malignant
31	2	1.5	Subsolid nodule	Malignant
32	2	1.1	Subsolid nodule	Malignant
33	3	1.5	Subsolid nodule	Malignant
34	7	1.6	Subsolid nodule	Malignant
35	12	1	Subsolid nodule	Malignant
36	3	1.4	Solid nodule	Malignant
37	3	0.8	Subsolid nodule	Malignant
38	2	1.3	Solid nodule	Malignant
39	4	1.4	Solid nodule	Malignant
40	0	0.5	Subsolid nodule	Malignant
41	0	0.9	Subsolid nodule	Malignant
42	0	1.2	Solid nodule	Malignant
43	3	1.9	Solid nodule	Malignant
44	3	1.9	Subsolid nodule	Malignant
45	5	1.3	Solid nodule	Malignant
46	5	1.3	Subsolid nodule	Malignant
47	12	2	Subsolid nodule	Malignant
48	0	2	Subsolid nodule	Malignant
49	4	0.7	Subsolid nodule	Malignant
50	5	1.3	Solid nodule	Malignant

51	5	1.4	Subsolid nodule	Malignant
52	2	2	Subsolid nodule	Malignant
53	0	1	Subsolid nodule	Malignant
54	3	2	Solid nodule	Malignant
55	6	1.8	Solid nodule	Malignant
56	0	1.3	Solid nodule	Malignant
57	2	1.9	Solid nodule	Malignant
58	2	1.9	Solid nodule	Malignant
59	40	1.5	Solid nodule	Malignant
60	3	1.7	Solid nodule	Malignant
61	6	1	Solid nodule	Malignant
62	9	1.9	Subsolid nodule	Malignant
63	10	1.8	Solid nodule	Malignant
64	14	1.1	Subsolid nodule	Malignant
65	4	1	Solid nodule	Malignant
66	0	2	Solid nodule	Malignant
67	6	1.2	Solid nodule	Malignant
68	2	1.5	Subsolid nodule	Malignant
69	2	1.9	Solid nodule	Malignant
70	2	1.5	Solid nodule	Malignant
71	7	1.8	Solid nodule	Malignant
72	47	2	Solid nodule	Malignant
73	1	1.8	Solid nodule	Malignant
74	8	1.7	Solid nodule	Malignant
75	2	1.6	Subsolid nodule	Malignant
76	4	1.3	Subsolid nodule	Malignant
77	1	1.1	Subsolid nodule	Malignant
78	11	1	Solid nodule	Malignant
79	7	0.8	Subsolid nodule	Malignant
80	3	1.2	Subsolid nodule	Malignant
81	0	1	Subsolid nodule	Malignant
82	2	1	Subsolid nodule	Malignant
83	40	1	Subsolid nodule	Malignant

84	3	1.5	Solid nodule	Malignant
85	5	1.6	Subsolid nodule	Malignant
86	7	1.2	Subsolid nodule	Malignant
87	6	1.6	Solid nodule	Malignant
88	6	1.2	Subsolid nodule	Malignant
89	4	2	Subsolid nodule	Malignant
90	1	1	Solid nodule	Benign
91	0	1.6	Solid nodule	Benign
92	1	1.2	Solid nodule	Benign
93	0	1.4	Solid nodule	Benign
94	0	2	Solid nodule	Benign
95	0	2	Solid nodule	Benign
96	1	0.7	Solid nodule	Benign
97	1	0.8	Subsolid nodule	Benign
98	0	1.1	Solid nodule	Benign
99	3	0.4	Solid nodule	Benign
100	0	1.6	Solid nodule	Benign
101	3	0.8	Subsolid nodule	Benign
102	1	1	Solid nodule	Benign
103	0	0.6	Solid nodule	Benign
104	0	2	Solid nodule	Benign
105	0	0.8	Solid nodule	Benign
106	12	1.5	Solid nodule	Benign
107	1	2	Solid nodule	Benign
108	1	1.8	Solid nodule	Benign
109	1	1.1	Subsolid nodule	Benign
110	1	1.1	Subsolid nodule	Benign
111	0	0.6	Subsolid nodule	Benign
112	6	1.5	Subsolid nodule	Benign
113	0	1.5	Solid nodule	Benign
114	1	2	Solid nodule	Benign
115	0	1.4	Solid nodule	Benign
116	3	1.1	Solid nodule	Benign

117	1	1.4	Subsolid nodule	Benign
118	1	1.9	Solid nodule	Benign
119	1	1.1	Solid nodule	Benign
120	0	2	Solid nodule	Benign

Supplyment Table 2. ROC analysis of CTCs in the pulmonary nodule patients with different diameter.

	All patients	≦1cm nodule	1-2 cm nodule
AUC	0.843	0.798	0.858
95%Cl	0.759-0.927	0.644-0.952	0.753-0.963
Best Cut-off Value	1.5	1.5	1.5
Sensitivity	0.8539	0.7727	0.8806
Specificity	0.8387	0.7778	0.8636
Negative Predictive Value	0.6667	0.5833	0.7037
Positive Predictive Value	0.9383	0.8947	0.9516
True Positive Rate	0.8539	0.7727	0.8806
False Positive Rate	0.1613	0.2222	0.1364
True Negatice Rate	0.8387	0.7778	0.8636
False Negative Rate	0.1461	0.22723	0.1194
False Discovery Rate	0.0617	0.1053	0.0484
Accuracy	0.85	0.7742	0.8764
Precision	0.9383	0.8947	0.9516
Youden Index	1.6926	1.5505	1.7442

Supplementary Table 3. Correlations between CTCs and tumor biomarkers.

	CTC correlation		
	Pearson r	95% Cl	p
CEA (n=86)	0.11	-0.108 to 0.312	0.33
NSE (n=89)	0.08	-0.127 to 0.287	0.44
pro-GRP (n=88)	-0.08	-0.282 to 0.135	0.48
CYRFA21-1 (n=89)	-0.03	-0.234 to 0.182	0.80
Diameter (n=89)	-0.02	-0.224 to 0.1193	0.88

Supplementary Table 4. ROC analysis of the number of CTCs, the expression levels of CEA, NSE, pro-GRP, and CYFRA21-1 in the pulmonary nodule patients enrolled.

	AUC	95% Cl	Accuracy	Precision	Youden Index	p
CTCs	0.843	0.759-0.927	0.85	0.9383	1.6926	/
CEA	0.524	0.412-0.637	0.4417	0.8929	1.1841	<0.0001
NSE	0.530	0.414-0.646	0.5	0.8537	1.1997	<0.0001
pro-GRP	0.502	0.375-0.629	0.7083	0.77	1.1232	<0.0001
CYFRA21-1	0.559	0.445-0.673	0.4333	0.8621	1.1519	0.0001
